# Behavioural Diversity Study in Bottlenose Dolphin (*Tursiops truncatus*) Groups and Its Implications for Welfare Assessments

**DOI:** 10.3390/ani11061715

**Published:** 2021-06-08

**Authors:** Fabienne Delfour, Ruta Vaicekauskaite, Daniel García-Párraga, Cristina Pilenga, Agathe Serres, Isabelle Brasseur, Ana Pascaud, Enrique Perlado-Campos, Guillermo J. Sánchez-Contreras, Katrin Baumgartner, Tania Monreal-Pawlowsky

**Affiliations:** 1 Parc Asterix, 60128 Plailly, France; 2 Marine Research Institute, Klaipeda University, 92294 Klaipeda, Lithuania; ruta@fox-zooconsulting.com; 3 Lithuania & Fox Consulting, 67500 Haguenau, France; 4 Fundación Oceanográfic de la Comunitat Valenciana, 46013 Valencia, Spain; dgarcia@oceanografic.org; 5 Zoomarine Italia, 00071 Pomezia, Italy; cpilenga@zoomarine.it; 6 Institute of Deep Sea Science and Engineering, Chinese Academy of Sciences, Sanya 572000, China; agathe.serres11@gmail.com; 7 Marineland Parks, CS 91111, CEDEX, 06605 Antibes, France; i.brasseur@marineland.fr; 8 ONIRIS—Ecole Nationale Vétérinaire de Nantes, 101 Route de Gachet, 44307 Nantes, France; pascaud.ana@gmail.com; 9 Planète Sauvage, 44710 Nantes, France; 10 Mundomar Benidorm, 03503 Benidorm, Spain; Kike@mundomar.es; 11 Mediterraneo Marine Park, NXR9038 Bahar ic-Caghaq, Malta; vet@mediterraneopark.com; 12 Zoo Nuremberg, Am Tiergarten 30, 90480 Nuremberg, Germany; Katrin.baumgartner@stadt.nuernberg.de; 13 International Zoo Veterinary Group, Keighley BD21 4NQ, UK; t.monreal@izvg.co.uk

**Keywords:** animal welfare, welfare assessment, welfare indicator, behavioural diversity, marine mammals, bottlenose dolphin

## Abstract

**Simple Summary:**

For the first time, a behavioural diversity study was conducted on several bottlenose dolphins *(Tursiops truncatus)* groups within European Association of Aquatic Mammals (EAAM) accredited facilities. This study was carried out by professional animal staff on 54 dolphins, and the goal was to analyse behavioural diversity in bottlenose dolphins at the group level to investigate how particular factors might impact the diversity of behaviours within the group and to discuss its implications for dolphin welfare assessments. This study showed its feasibility and revealed impacting factors that would need to be considered in future dolphin welfare assessments. We strongly believe that behavioural evaluations and measurements could be applied routinely on cetaceans under professional care to assess their welfare.

**Abstract:**

In the recent past, animal welfare studies have tried to determine the best animal welfare measures and indicators. Expression of behavioural diversity is considered a potential positive welfare indicator, and to the authors’ knowledge, it has not been validated nor studied in cetaceans. For the first time, a behavioural diversity study on bottlenose dolphins *(Tursiops truncatus)* groups was conducted at six European facilities. The study was carried out by the animal care staff, biologists and veterinarians and included 54 dolphins housed in several group compositions at the different participating facilities. The goal of our study was to analyse behavioural diversity in bottlenose dolphins at the group level to investigate how particular factors might impact the diversity of behaviours within the group and to discuss its implications for dolphin welfare assessments. Eight factors (i.e., “observer location”, “number of individuals”, “age class”, “sex”, “social grouping”, “presence/absence of leading male”, “presence/absence of visitors” and “enrichment provision”) impacted the behavioural diversity of the observed groups, while no significant impact of the factors “time of day” and “activity before/after observation” could be found. Our study showed the feasibility of this kind of approach for cetaceans under professional care and the relevance to considering this parameter in dolphin welfare studies, despite certain limitations that warrant further research.

## 1. Introduction

Behaviour is one of the keystones when assessing animal welfare, together with cognition (i.e., mental states and emotions) and physiology (i.e., health) [1]. These three pillars contribute to modernising initial animal welfare definitions, too often limited to the presence of negative behaviours, such as frequent aggressive behaviours or self-injurious activities. This current approach also requires combining resource- and animal-based indicators to accurately measure the animal’s welfare state. If, in the past, animal welfare assessments mostly relied on negative indicators, scientists now also look for positive indicators [2]. In the actual holistic approach of animal welfare, it is commonly accepted to conduct a variety of measurements using both negative (e.g., apathy, social isolation, inappetence and lameness) and positive (e.g., play, exploratory behaviour and social affiliative behaviour) welfare indicators. One current positive welfare indicator is related to the richness of the animals’ behavioural repertoire. The rationales are that an animal displaying various behaviours is in a better welfare state than an individual showing a limited behavioural repertoire, and that in the case where an animal is unable to perform a behaviour it is motivated to display, its welfare is diminished [3].

In other words, behavioural diversity is considered to be a positive welfare indicator since it could be lost or impaired when the individual has to face challenging situations. In the scientific literature, this parameter is differently named: behavio(u)ral diversity, etho-diversity or behavio(u)ral variety [4,5,6]. It describes the richness and the evenness of displayed behaviours. Miller et al. (2020) [7] reviewed ways to evaluate behavioural diversity. When working with zoo animals, the authors listed several factors influencing behavioural diversity, such as environmental enrichment, habitat complexity, social grouping and animal training. The authors also pinpointed very relevant limitations to consider when working with behavioural diversity indicators, such as the assumptions that all behaviours are equally important for the animals to perform or that all behaviours are equally identifiable and detectable.

Social animals display behavioural diversity due to genetic and/or social evolutionary processes influenced by the environment (Whitehead et al., 2019) [8]. In social insect societies group behavioural diversity has been linked to individual fitness and group success (e.g., aggressive honeybee colonies show a high winter survival rate and docile social spiders grow faster than their aggressive conspecifics) (see Modlmeier et al., 2014) [9]. Group behavioural diversity has also been studied in primates. Using all occurrence hunting behaviours, Samuni et al. (2020) [10] documented bonobos’ hunting and feeding patterns. Kühl et al. (2019) [11] studied 31 specific behaviours in 144 communities of chimpanzees and showed that group behavioural diversity was decreased when wild chimpanzees lived in areas with high human impact. The authors used presence and absence data on those 31 known behaviours from 46 chimpanzee communities and complemented their data set with additional information on another 106 chimpanzee groups using published scientific literature. They then completed their study by showing that wild chimpanzees communities exhibit greater behavioural diversity in environments with more variability (Kalan et al., 2020) [12]. Studying group behavioural diversity is a parameter to consider in conservation management when examining the combined effects of ecology, habitat, demography and phylogeny on behaviour (Boesh et al., 2002) [13]. Moreover, group behavioural diversity has been studied in six zoo wolf packs to understand their overall welfare (Frézard and Le Pape, 2003) [14]. The authors analysed the groups’ behavioural differences according to restrictive or more permissive living conditions and found that the size of the enclosure did not impact group behavioural diversity much, but the composition of the pack did. They then suggested using their results to optimize the wolves’ welfare.

In zoological park settings, welfare assessments have become more frequent because of general public concerns and zoo professionals emphasising the need to measure the welfare of their animals objectively and scientifically [15]. Several of those assessments include and use behavioural diversity indexes in a variety of zoo animal species: for instance, in elephants (Loxodonta africana, Elephas maximus) [16], reptiles [17,18], cheetahs (Acinonyx jubatus) [19], gentoo penguins (Pygoscelis papua) [20], flamingos (Phoenicopteridae) [21], lions (Panthera leo) [22], aardvarks (Orycteropus afer) [23] and red foxes (Vulpes vulpes) [24], among others. Studies showed that behavioural diversity is influenced by environmental enrichment [7,25], as described in African lions (Panthera leo) [26], in Australian fur seals (Arctocephalus pusillus doriferus) [27] and in European wolves (Canis lupus lupus) [28]. Social grouping and animal training also impact behavioural diversity [7,29]. There is a need for validated measures of positive animal welfare, and there is a growing body of evidence that supports the use of behavioral diversity as a positive indicator of welfare. This includes an inverse relationship with stereotypic behavior as well as faecal glucocorticoid metabolites and is typically higher in situations thought to promote positive welfare [7].

Marine mammals under professional care have been subjects of several animal welfare studies [30] with good examples in harbour seals (Phoca vitulina) [31], California sea lions (Zalophus californianus) [32], polar bears (Ursus maritimus) [33,34] and beluga whales (Delphinapterus leucas) [35]. More studies have now been conducted on bottlenose dolphins [36]. Researchers have analysed the effects of human-controlled period schedules [37]; participation in dolphin–human interaction programs [38] and in education programs [39], environmental changes and anthropogenic factors [40]; body contact and social interactions [41]; dolphins’ cortisol salivary level [42,43,44]; and their willingness to participate in training sessions [43,44]. Several positive welfare indicators have been validated and/or deserve further investigation: synchronous swimming [44,45], willingness to participate in trainings [45,46], social interactions and body contact [47,48], social play [40] and anticipatory behaviour [49,50]. During the last decades, dolphinaria have been working on improving animal welfare in their habitats, including specific environmental enrichment programs in alignment with European legislation [51] and international WAZA recommendations [15]. It is generally assumed that enrichment creates opportunities for animals to display a variety of behaviours and encourages affiliative behaviours (e.g., positive social interactions) and play behaviours [52]. Environmental enrichments are usually considered to be of five types: structural, nutritional, sensorial, social and cognitive, and all five have been used with dolphins [53].

In our study, we aimed to analyse behavioural diversity in bottlenose dolphins at group level to investigate how particular factors might impact the diversity of behaviours within the group. Following the scientific literature, we investigated the influence of ten different factors (i.e., time of day, the observer location, the number and sex composition of the group, the age of the individuals, the social grouping, the presence of a leading male, the activity before and after the observation, the presence of visitors and the presence of enrichment devices) on six main behavioural categories (i.e., affiliative behaviours, agonistic behaviours, solitary behaviours, play/exploratory behaviours, sexual behaviours and maternal behaviours).

## 2. Materials and Methods

This study adhered to the ASAB/ABS Guidelines for the Use of Animals in Research. This study was conducted in accordance with the Declaration of Helsinki.

### 2.1. Study Sites and Subjects

From the end of October 2020 to mid-December 2020, we studied 54 bottlenose dolphins (Tursiops truncatus) housed in six European dolphinaria: two in France, two in Spain, one in Malta and one in Italy. The animals were divided into nine subgroups and were constituted by 29 females and 25 males, ranging from 7 months to 40 years old.

### 2.2. Assessment of Behavioural Diversity

To investigate the behavioural diversity within our dolphin groups, we reviewed present scientific literature, we followed Miller et al.’s (2020) [7] recommendations and we decided to revisit the methods used by Spiezo et al. (2018) [6], Kühl et al. (2019) [11] (i.e., presence/absence of behaviours) and Frézard and Le Pape (2003) [14] (i.e., regular group scan observations on several days). We evaluated groups and discussed the results in light of animal welfare. Spiezo et al. [6] grouped together various behaviours into specific and already well-defined categories according to their behavioural functions (e.g., affiliative, agonistic) and Frézard and Le Pape [14] mixed behavioural items (e.g., sitting, sleeping) and behavioural categories (e.g., negative social with 8 behavioural items). However, here we followed Spiezo et al.’s [6] methodology and we selected dolphins’ behaviours based on previous scientific literature. We created a list of 55 behaviours of interest grouped into six categories (five to nineteen behaviours in each category) based on their behavioural functions (Table 1). A definition for each behaviour was included and sent to the participating dolphinaria. In order not to influence the observers when noting the behaviours, only the list of the behaviours and their definition without the corresponding classified categories were distributed.

### 2.3. Behavioural Sampling

During continuous observation sessions (N = 40/subgroup) lasting 15 min each, an experienced trainer, marine mammal biologist or veterinarian (i.e., more than 5 years of experience working with dolphins) recorded the occurrence of behaviours of interest using a focal group sampling method [54] (i.e., the observer noted when one or more animals within the observed group displayed one or more behaviours of the defined repertoire).

The authors are aware of the interobserver variability. However, all the persons involved in conducting the observations were familiar with behavioural studies and had already been involved in several research projects in the past.

Depending on each dolphinarium design, the observer could choose to conduct his/her observations above the water surface and/or with an under water view (i.e., “observer location”). Whilst the location of an observer does not directly affect dolphins’ welfare, observer location may be methodologically relevant to measuring dolphin welfare, hence it was included in the statisticial analysis. At each dolphinarium, 40 sessions were recorded/subgroup: 10 sessions in the morning (until 11 am), 10 during midday (between 11 a.m. and 2:30 p.m.) and 20 in the afternoon (after 2:30 p.m.) (i.e., “time of day”). Of these 20 afternoon sessions, 10 were without enrichment devices in the pools and 10 included enrichment objects (i.e., “enrichment provision”). The impact of five types of enrichment was analysed: structural, cognitive, nutritional, sensorial and mixed (i.e., two previous types combined). For standardization of the study, enrichment occurred only during afternoon sessions. For each session, the “number of individuals” in the subgroup was recorded as well as the “age class” (i.e., “juveniles”, “adults” or “adults + juveniles”). For husbandry reasons, juveniles were never separated from their adult affiliates. The sex of the subgroup was also noted (i.e., “males”, ”females” or “mixed sex group”). For our study, we considered two social groupings (i.e., factor “social grouping”): some of the observed subgroups within the same institution were separated from others for a time period longer than five days, and it was considered as “long separation” for our study, while in some other instances, all the animals were kept “together” forming a single unit subgroup. The “presence/absence of leading male” and the “presence/absence of visitors” were also considered as potential influencing factors and recorded for each observation session. Some facilities were open at the time of the study, and visitors could access the dolphinarium to freely look at the dolphins. Finally, the “activity before” and the “activity after” observation were also documented as “free time”, “training” or “public presentation”. Data were collected during the animals’ free time, with no primary reinforcement and without any medical, training and/or feeding activities.

### 2.4. Statistical Analysis

Statistical analyses were done with the programme R, version 4.0.3 [55]. Behavioural diversity is defined here as the number of different behaviours observed in total and for each behavioural category during each observation session. The behavioural diversity in this study was analysed using generalised linear mixed effects models (GLMMs), with the “glmmTMB” function from the “glmmTMB” package [56].

As behavioural diversity variables were numerical count data, models were fitted for “Poisson” distributed data (log link). For all models, we included the identity of the dolphinaria and the observation day as random factors to account for nested measurements. Predictors included the time of day (i.e., morning, midday or afternoon), the observer location (i.e., underwater, above water or both), the size of the group (from 2 to 9), the group sex composition (i.e., only males, only females or both sexes), the group age composition (i.e., only adults or juveniles and adults), the social grouping (i.e., individuals all together or separated), the presence of a leading male (yes or no), the activity before the observation and the activity after the observation (i.e., free-time, training, public presentation or enrichment), the presence of visitors around pools (yes or no) and the presence of enrichment (i.e., none, structural, cognitive, nutritional, sensorial or mixed). During our study, no social enrichment (i.e., to promote and/or to enhance intraspecific social interactions) was provided in any of the participating dolphinaria. In order to account for the effect that the number of mother–calf pairs presents on the diversity of maternal behaviours, this variable was added as a predictor to the “maternal behavioural diversity” model.

The number of individuals present allowed for accounting for stochastic size effects. A total of seven models were run with the response variables being (1) the total behavioural diversity, (2) the affiliative behavioural diversity, (3) the agonistic behavioural diversity, (4) the solitary behavioural diversity, (5) the play/exploratory behavioural diversity, (6) the sexual behavioural diversity and (7) the maternal behavioural diversity. Over dispersion and collinearity were checked and revealed no problems, an additional residuals distribution diagnosis was conducted using the “DHARMa” package [57] and model selection was achieved using the “MuMIn” Package [58]. For each response variable, among all tested models, the one with the lowest Akaike information criterion [59] was selected. Selected models could contain nonsignificant variables, including the number of individuals present, which were always used in the models to account for group size effect (i.e., the probability of seing any particular behaviour increases with the number of individuals present). Wald chi-squared tests were used to extract *p*-values from models. Post hoc tests were achieved by running the selected models with appropriate subsettings, and a sequential Bonferroni correction was applied to the outputs.

## 3. Results

### 3.1. Total Behavioural Diversity

**Figure 1:**
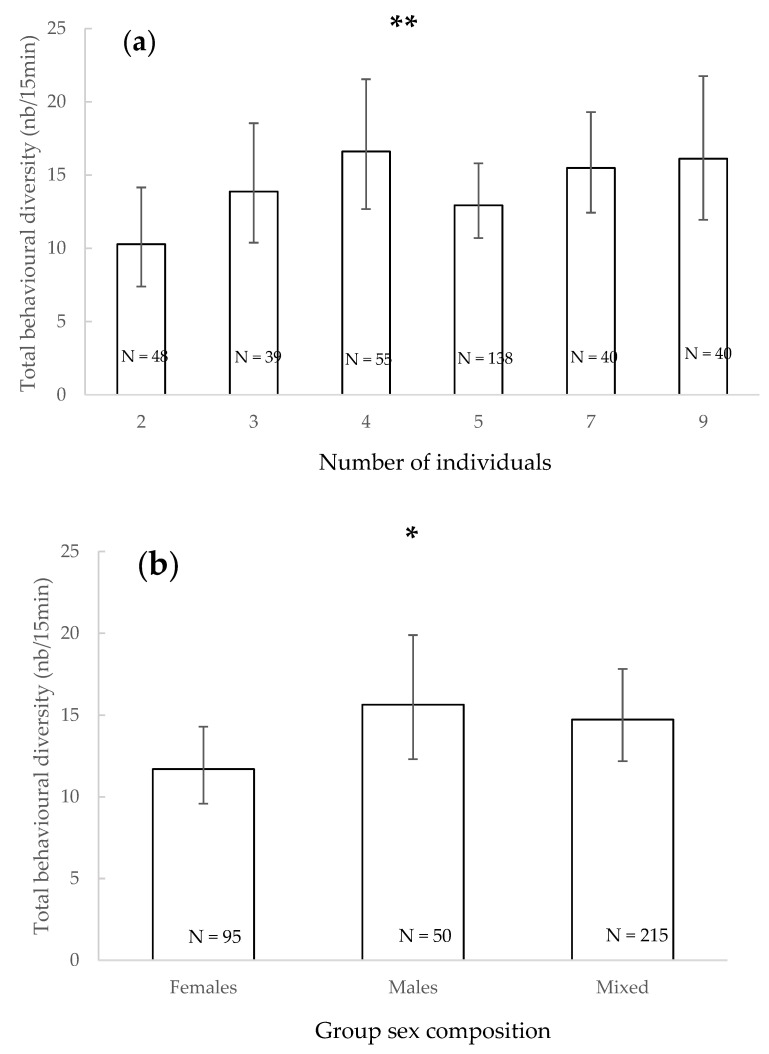

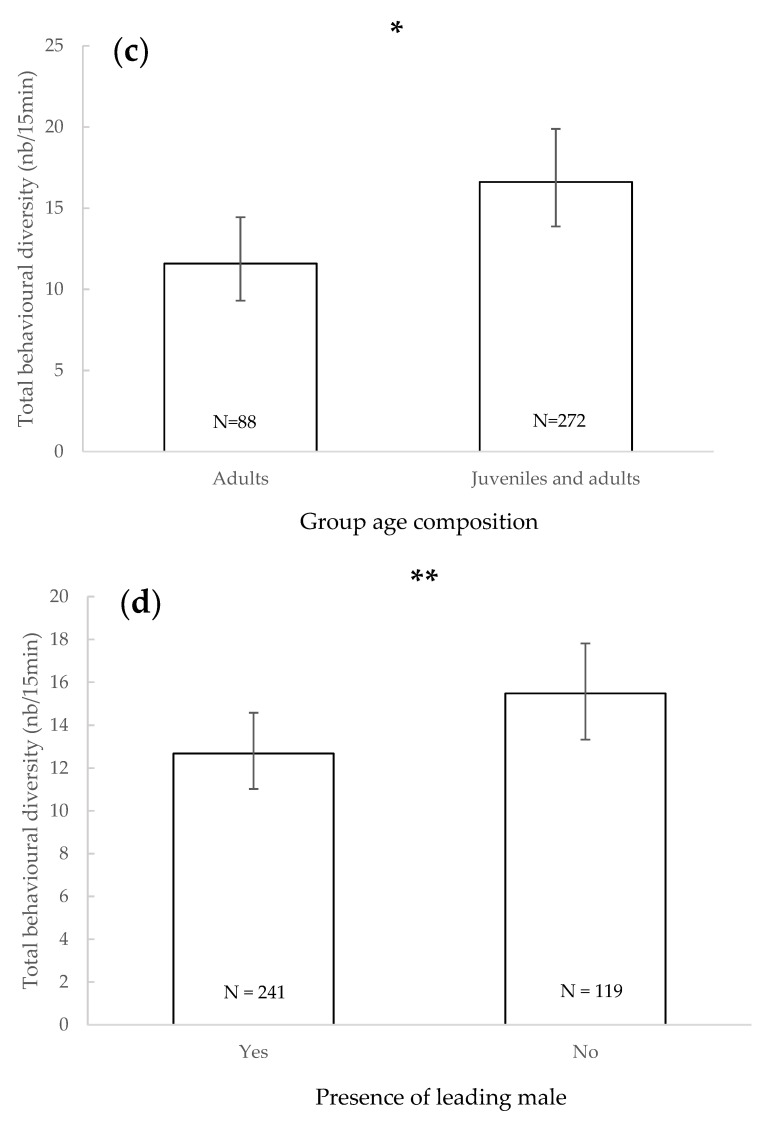
Mean of total behavioural diversity and 95% confidence intervals according to the number of individuals present (**a**), the group sex composition (**b**), the group age composition (**c**) and the presence of the leading male (**d**). *: *p* < 0.05, **: *p* < 0.01 (Wald chi-squared test). N: number of observations.

The total variety of behaviours observed during observation sessions significantly increased with the number of individuals present (χ^2^ = 9.748, df = 1, *p* = 0.002, Figure 1a). The sex of the individuals significantly impacted the total behavioural diversity (χ^2^ = 6.379, df = 2, *p* = 0.041), but pairwise tests did not reveal significant differences between each sex composition condition (Figure 1b). The total variety of behaviours was significantly higher when both juveniles and adults were present than with adults only (χ^2^ = 5.888, df = 1, *p* = 0.015, Figure 1c), and it was significantly higher when the leading male was absent than when he was present (χ^2^ = 7.510, df = 1, *p* = 0.006, Figure 1d). Other factors did not significantly impact the total diversity of behaviours observed (*p* > 0.05).

### 3.2. Affiliative Behavioural Diversity

**Figure 2:**
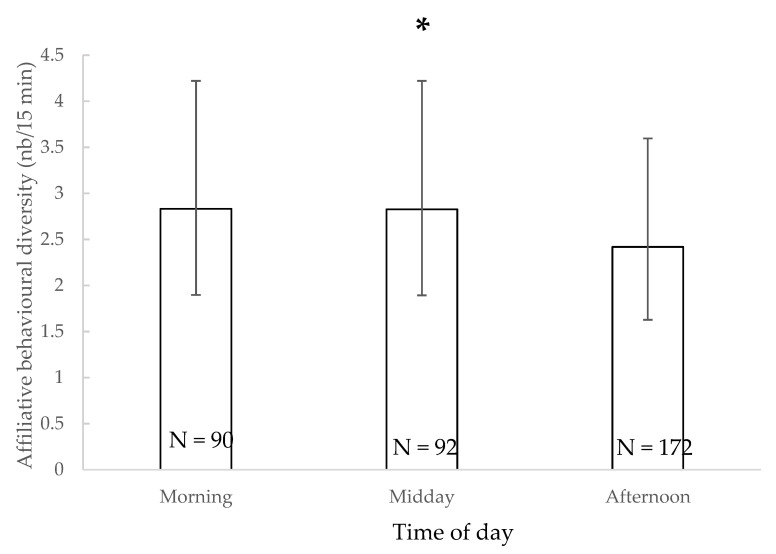
Mean of affiliative behavioural diversity and 95% confidence intervals according to the time of day. *: *p* < 0.05 (Wald chi-squared test). N: number of observations.

The variety of affiliative behaviours observed during observation sessions was significantly impacted by the time of day (χ^2^ = 7.839, df = 2, *p* = 0.019), but pairwise tests did not reveal significant differences between each time period (Figure 2). Other factors did not significantly impact the diversity of affiliative behaviours observed (*p* > 0.05).

### 3.3. Agonistic Behavioural Diversity

**Figure 3:**
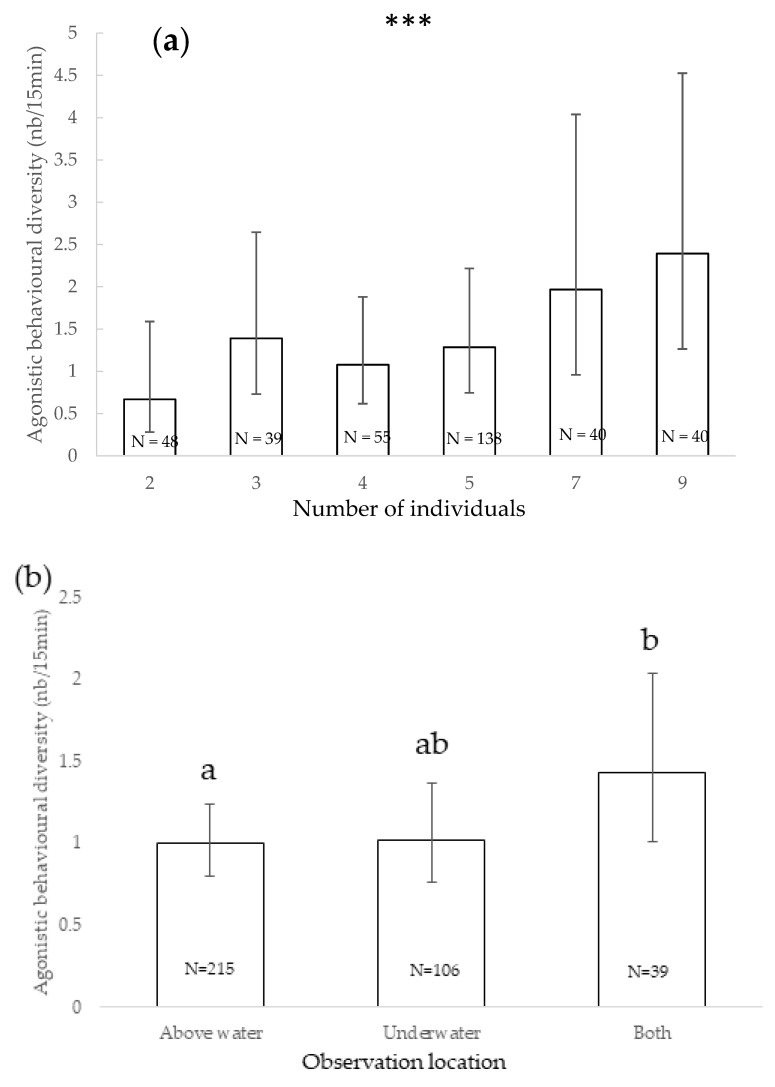

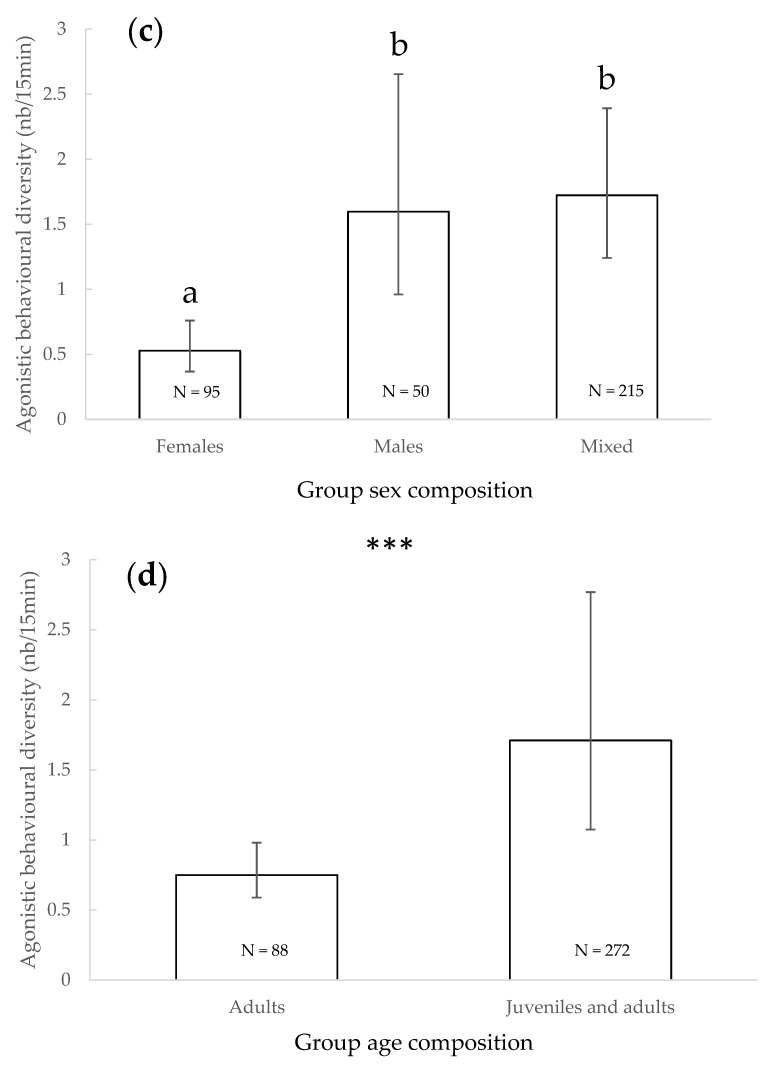

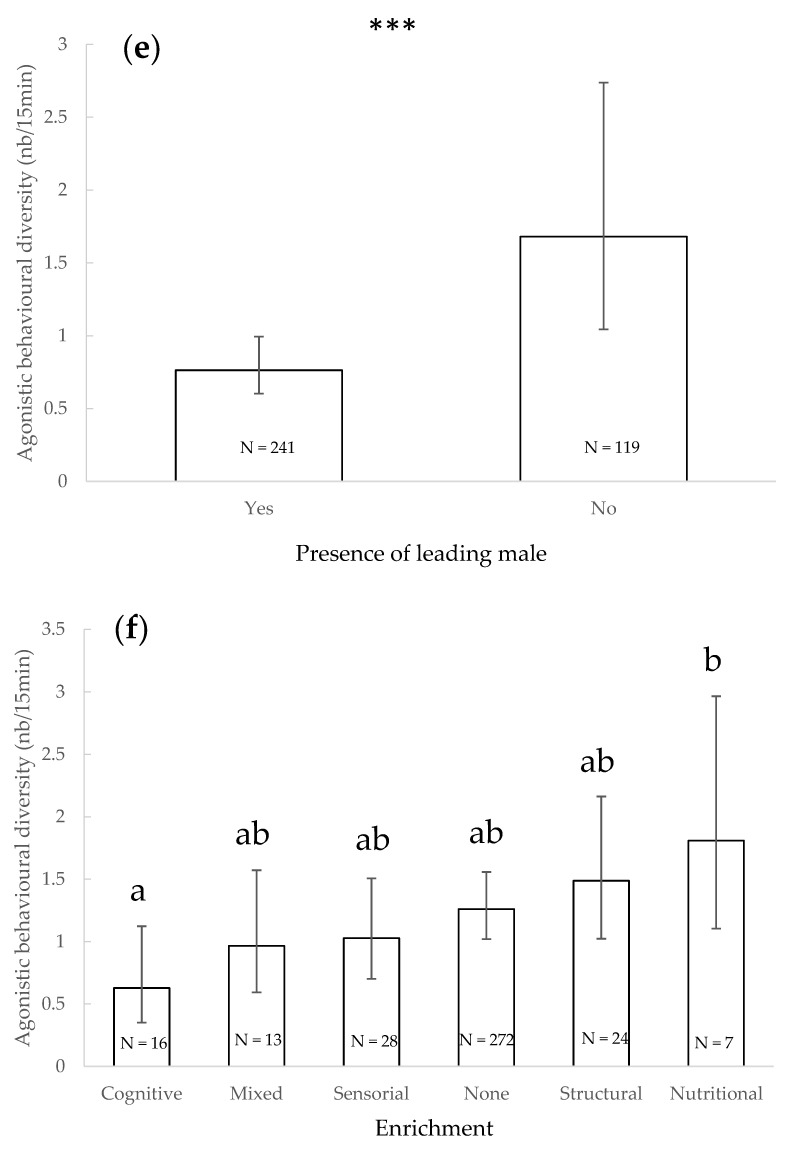
Mean of agonistic behavioural diversity and 95% confidence intervals according to the number of individuals (**a**), observation location (**b**), sex of the individuals (**c**), age of the individuals (**d**), presence of leading male (**e**) and presence of enrichment (**f**). ***: *p* < 0.001; within each factor, categories that share the same letter do not differ significantly and categories that have no letter in common differ significantly or tend to differ (Wald chi-squared test with sequential Bonferroni correction). N: number of observations.

The variety of agonistic behaviours observed during observation sessions significantly increased with the number of individuals present (χ^2^ = 16.366, df = 1, *p* < 0.001) (Figure 3a). The diversity of agonistic behaviours observed during observation sessions was also significantly impacted by the location of observation (χ^2^ = 16.366, df = 2, *p* < 0.001): it tended to be higher when observations were conducted both under and above water than when conducted above water only (*p* = 0.031) (Figure 3b). The sex of the individuals significantly impacted the agonistic behaviours diversity (χ^2^ = 32.703, df = 2, *p* < 0.001): it was significantly higher when only males were present (*p* = 0.001) or when both sexes were present (*p* < 0.001) than when only females were (Figure 3c). The variety of agonistic behaviours was significantly higher when both juveniles and adults were present than with adults only (χ^2^ = 11.453, df = 1, *p* < 0.001) (Figure 3d), and it was significantly higher when the leading male was absent than when he was present (χ^2^ = 20.362, df = 1, *p* < 0.001) (Figure 3e). The presence of enrichment also significantly impacted the diversity of agonistic behaviours observed (χ^2^ = 12.249, df = 5, *p* = 0.032): it tended to be higher when nutritional enrichment was provided than when cognitive enrichment was present (*p* = 0.044, Figure 3f). Other factors did not significantly impact the agonistic behaviours diversity (*p* > 0.05).

### 3.4. Solitary Behavioural Diversity

**Figure 4:**
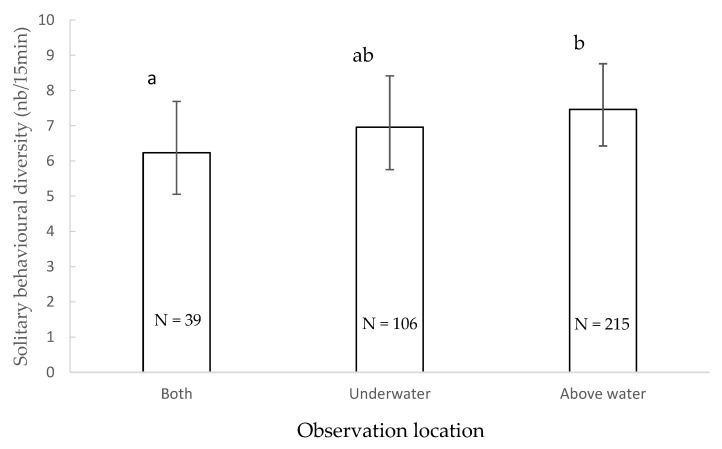
Mean of solitary behavioural diversity and 95% confidence intervals according to the observation location. Locations that share the same letter do not differ significantly and locations that have no letter in common tend to differ (Wald chi-squared test with sequential Bonferroni correction). N: number of observations.

The diversity of solitary behaviours observed during observation sessions was significantly impacted by the location of observation (χ^2^ = 6.221, df = 2, *p* = 0.045) (Figure 4): it tended to be higher when observations were conducted above water only than when conducted both under and above water (*p* = 0.043). Other factors did not significantly impact the total diversity of behaviours observed (*p* > 0.05).

### 3.5. Play/Exploratory Behavioural Diversity

**Figure 5:**
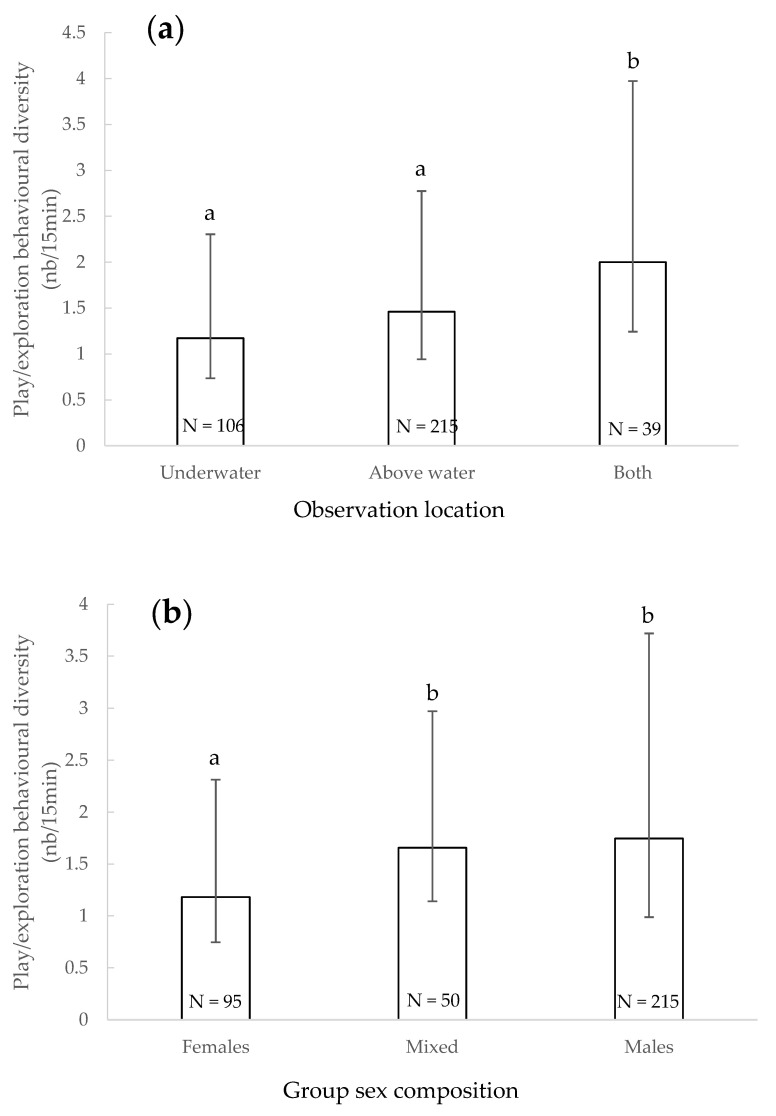

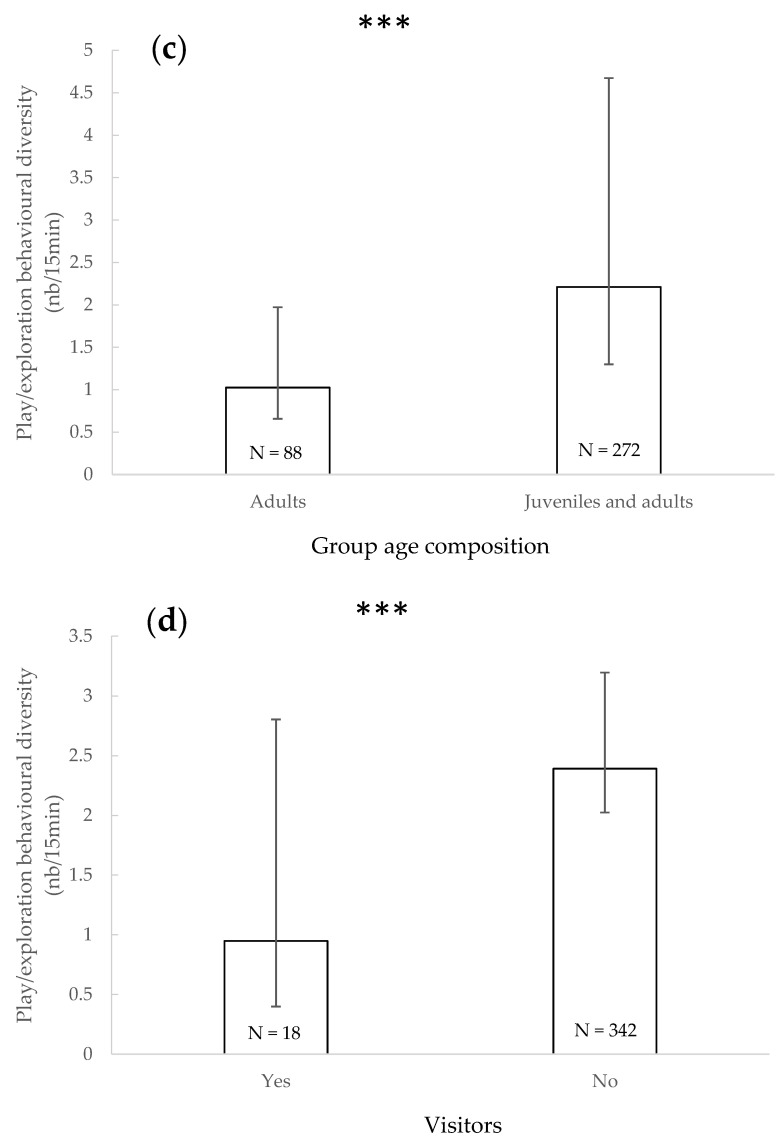

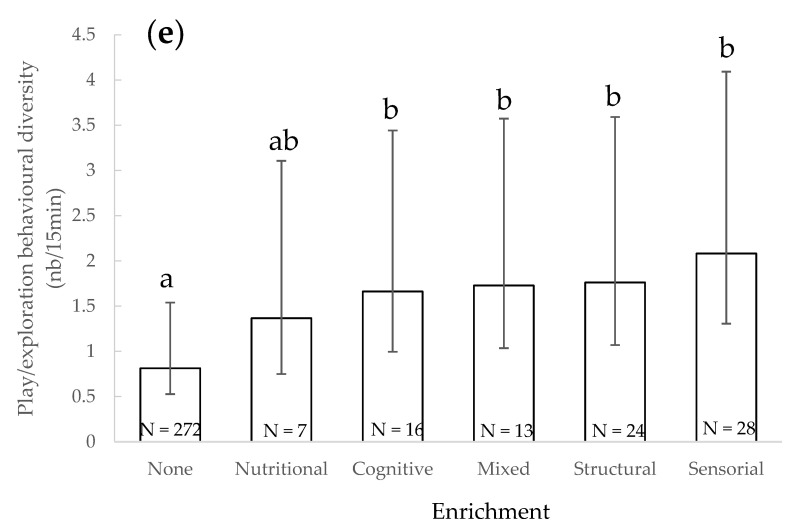
Mean of the play/exploratory behavioural diversity and 95% confidence intervals according to the observation location (**a**), sex of the individuals (**b**), age of the individuals (**c**), presence of visitors (**d**) and presence of enrichment (**e**). ***: *p* < 0.001; within each factor, categories that share the same letter do not differ significantly and categories that have no letter in common differ significantly or tend to differ (Wald chi-squared test with a sequential Bonferroni correction). N: number of observations.

The diversity of play/exploratory behaviours observed during observation was significantly impacted by the location of the observer (χ^2^ = 22.975, df = 2, *p* < 0.001) (Figure 5a): it was significantly higher when observations were conducted both under and above water than when conducted above water only (*p* = 0.017) or underwater only (*p* < 0.001). The sex of the individuals significantly impacted the play/exploration behaviours diversity (χ^2^ = 8.908, df = 2, *p* = 0.012) (Figure 5b): it was significantly higher when both sexes were present (*p* < 0.001) and tended to be higher when only males were present (*p* = 0.022) than with only females. The variety of play/exploration behaviours was significantly higher when both juveniles and adults were present than only with adults (χ^2^ = 12.539, df = 1, *p* < 0.001) (Figure 5c), and it was significantly higher when visitors were absent than when they were present (χ^2^ = 4.276, df = 1, *p* = 0.039) (Figure 5d). The presence of enrichment also significantly impacted the diversity of play/exploration behaviours observed (χ^2^ = 119.553, df = 5, *p* < 0.001) (Figure 5e): it was significantly higher when sensorial (*p* < 0.001), structural (*p* < 0.001), cognitive (*p* < 0.001) or mixed enrichment (*p* < 0.001) was provided than when no enrichment was present. Other factors did not significantly impact the total variety of behaviours observed (*p* > 0.05).

### 3.6. Sexual Behavioural Diversity

**Figure 6:**
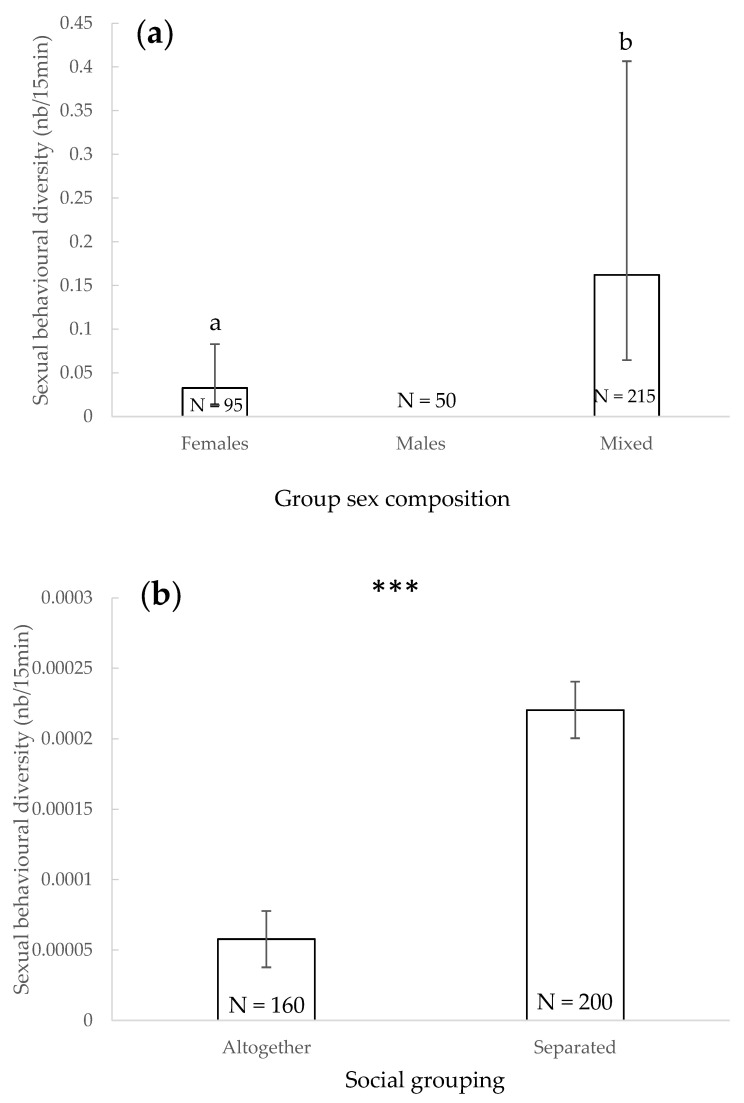
Mean of the sexual behavioural diversity and 95% confidence intervals according to the sex composition (**a**) and social grouping (**b**). ***: *p* < 0.001; within each factor, categories that share the same letter do not differ significantly and categories that have no letter in common differ significantly (Wald chi-squared test with a sequential Bonferroni correction). N: number of observations.

The diversity of sexual behaviours observed during an observation session was significantly impacted by the sex composition (χ^2^ = 12.449, df = 2, *p* = 0.002) (Figure 6a): it was significantly lower when only females were present than when both sexes were included in the study group (*p* = 0.001). The diversity of sexual behaviours was significantly higher if a lasting separation between subgroups of animals was occurring in that particular facility than when animals were maintained altogether (χ^2^ = 18.518, df = 1, *p* < 0.001 (Figure 6b). Other factors did not significantly impact the total variety of sexual behaviours observed (*p* > 0.05).

### 3.7. Maternal Behavioural Diversity

**Figure 7:**
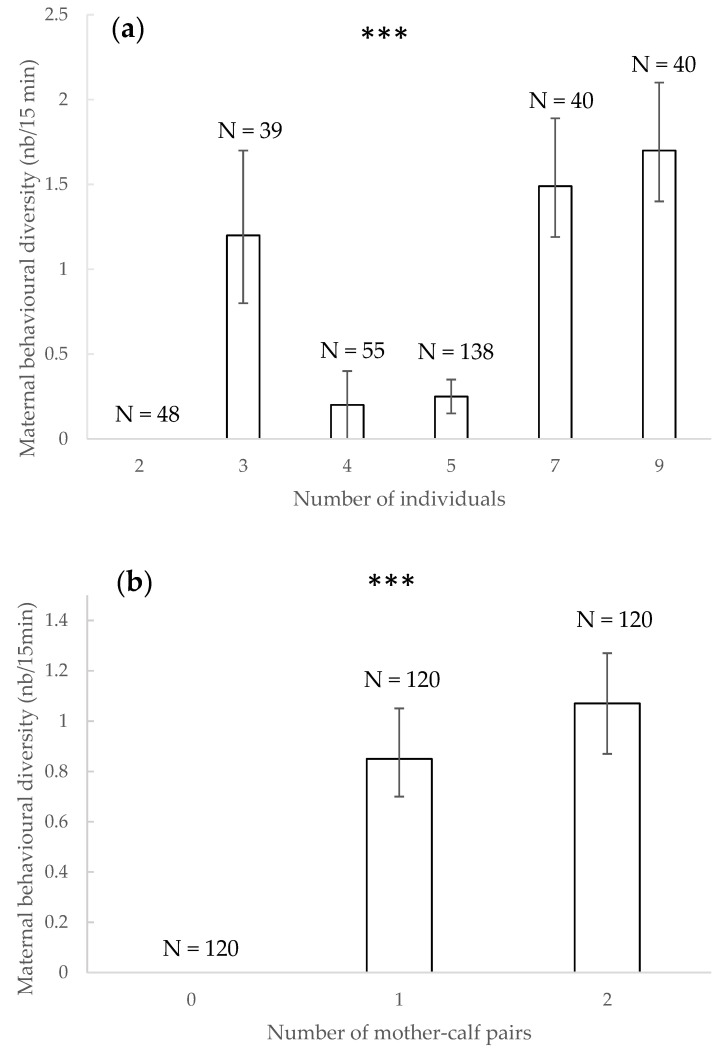
Mean of maternal behavioural diversity and 95% confidence intervals according to the number of individuals present (**a**) and number of mother–calf pairs present (**b**). ***: *p* < 0.001 (Wald chi-squared test). N: number of observations.

The diversity of maternal behaviours observed during an observation session significantly increased with the number of individuals present (χ^2^ = 13.091, df = 1, *p* < 0.001) (Figure 7a) and with the number of mother–calf pairs present (χ^2^ = 57.840, df = 1, *p* < 0.001) (Figure 7b).Other factors did not significantly impact the total diversity of maternal behaviours observed (*p* > 0.05).

## 4. Discussion

Agonistic and play/exploratory behavioural diversities appeared to be the categories impacted by a greater number of factors (i.e., six and five factors, respectively). The overall behavioural diversity of the dolphins was influenced by three factors (i.e., number of individuals, group age composition and presence/absence of leading male). Both sexual and maternal behaviours were impacted by two factors each. The dolphins’ affiliative and solitary behavioural categories were influenced by one factor (i.e., the time of day and observer location, respectively). The factor “activity before or after observation” had no impact on any of the behavioural diversity categories. This synthesis helps with understanding when and where observation sessions should be conducted when assessing dolphins’ welfare under professional care and will be further discussed.

The Dolphins’ behavioural diversity increased with the size of the group. The smallest group size during our study comprised two individuals, and the largest included nine individuals. An increase in size of the study group means a greater diversity in dolphins’ sex, age and personality but also in life histories and experiences, and consequently, this increased diversity amplifies behavioural diversity [60,61,62]. This result could seem quite obvious as Tursiops is a social delphinid (Tursiops sp. average group size ranges from 2 to 15 [63,64]); however, in animal groups where the majority of the individuals have very poor welfare, we might find a low group behavioural diversity.

Overall dolphins’ behavioural diversity was greater in groups with juveniles and adults than in adult only groups. Juveniles and adult groups displayed a great variety of behaviours covering the six categories we studied, including play and maternal behaviours, behaviours that might be less displayed (in time and occurrences) in adult only groups. Juveniles are also more energetic than adults, which could increase the group behavioural diversity [65]. Additionally, in the absence of a leading male, the dolphins’ behavioural diversity was greater than when he was present. The presence of the leading male might cause the group to settle down [66,67].

### 4.1. Affiliative Behavioural Diversity

Affiliative behaviours diversity was impacted by the time of day with a tendency to be greater in the mornings and middays vs. in the afternoons. According to previous studies focusing on particular behaviours, the factor time of day is relevant to consider when setting up a protocol to assess dolphins’ welfare [40]; morning and/or midday observations should be preferred over afternoon observations.

### 4.2. Agonistic Behavioural Diversity

The diversity of agonistic behaviours was greater when observations were conducted both under and above water, or only above water, when compared to only underwater. Considering the short durations of certain behaviours (e.g., bites, hits/bumps and jaw claps), this might make it more difficult to observe them from a limited underwater viewpoint. This suggests that certain observations are not accurate when performed only from one point of view, and this should be considered when performing dolphin welfare assessments both in the wild and under human care. Moreover, agonistic behaviours are usually energetic and create water swirls easily observable from the surface, but not so evident from an underwater perspective.

Mixed-sex groups and male groups showed greater agonistic behavioural diversity compared to only female groups. Males tend to display more agonistic and aggressive behaviours than females as part of intrasexual competition and intersexual conflicts (e.g., sexual coercion) [65], and some agonistic behaviours (e.g., chase, pivot dive) are part of the sexual behavioural patterns [67]. Agonistic behavioural diversity was greater in groups including juveniles and adults than in adult only groups. Juveniles are more energetic than adults, they display rough play behaviours involving some agonistic behaviours (e.g., chase, bite, pivot dive, hit) and they also challenge themselves and adults alike [65]. Disciplinary behaviours from adults to juveniles also involve chase, pivot dive and hitting behaviours, for instance [68], resulting in further displays of agonistic behaviours. In the absence of a leading male, the dolphins’ agonistic behavioural diversity was greater than in its presence. With the well-established leadership of one male, dolphins do not challenge each other to lead the group, resulting in fewer agonistic behaviours displayed [65].

Nutritional enrichment elicited more agonistic behavioural diversity than cognitive enrichment. Food involved in the first type of enrichment might have generated some competition, while cognitive enrichment for dolphins often consists in cooperative tasks or promotes solitary events [69,70]. Fast swimming also increased in presence of enrichment [41]. Different types of enrichment have already been seen to elicit different frequency variations in agonistic behaviours. The presence of humans and toys together resulted in a significantly lower frequency of agonistic interactions, whereas the presence of toys or humans alone did not decrease this frequency [41]. However, the opposite was found in another species included in a later study: Yangtze finless porpoises (Neophocaena phocaenoides) exhibited a higher frequency of agonistic behaviours when humans and toys were present at the same time, but not when toys or humans were present alone. The competition for enrichment may therefore vary depending on the type of enrichment, human presence and the species itself.

### 4.3. Solitary Behavioural Diversity

Observations conducted above water were able to identify more diverse solitary behaviours than observations conducted both above and underwater. This category gathered a consequent number of both aerial and underwater behaviours, but aerial behaviours displayed by one solitary animal are probably more noticeable from above the water surface (i.e., easy to spot breaking surface behaviours) than from underwater, where the whole group is and which is subjected to limitations such as narrow field view and visibility issues. We can also question whether from above water, the observers could have actually missed the presence of conspecifics swimming deeper in the pool, potentially leading to the classification of these as solitary behaviours that were not actually solitary. This again shows that in order to undertake a welfare assessment for cetaceans, it would be best to have access to underwater viewing as well as above water observations simultaneously or sequentially.

### 4.4. Play/Exploratory Behavioural Diversity

Dolphins’ play/exploratory behavioural diversity was impacted by five factors. The diversity was significantly higher when observations were conducted both under and above water than when conducted above water only or underwater only. Again, this strengthens our belief that observing from both points of view (simultaneously and/or sequentially) is essential to obtain reliable information on groups’ behaviours.

Play/exploratory behavioural diversity was also significantly higher when only males were present or when both sexes were present than with only females. Male dolphins tend to display more play behaviours than females [71,72]. However, dolphins’ personality should also be a factor to explore in future studies to understand if some individuals are more curious, extroverted and playful than others [60].

The play/exploratory behaviours index was significantly greater when both juveniles and adults were present than with only adults. It is important to note that if young dolphins play more (or have a higher play behavioural diversity) than adults, having young dolphins in a group will increase behavioural diversity, even if adult behaviour stays exactly the same (e.g., is not influenced by the presence of younger individuals). Juvenile bottlenose dolphins tend to be more energetic, extroverted, curious and playful than adults [60,73,74]. Adult dolphins imitate their young conspecifics [75], hence when both adult and young animals are present, the adult ones behave in a more youthful way by imitating the younger ones. Moreover, Hill and Ramirez (2014) [76] showed that in beluga whales (Delphinapterus leucas), adults tend to play more with objects than immatures, who tend to display more locomotor play than their adult conspecifics. Similar results have been found in bottlenose dolphins where adults engaged in more solitary object play than young animals [71]. In our study we did not distinguish forms of play, hence we cannot know if, when juveniles and adults were together, they displayed social play or if they displayed their distinct play and exploratory behaviours, increasing the diversity of this category. The nature of the play behaviour should be further investigated in future studies.

Dolphins’ play and exploratory behavioural diversity was greater when visitors were absent compared to when they were present. Social play in bottlenose dolphins increases in absence vs. presence of visitors [41]. This result could be due to visitors distracting dolphins from their play activities (we do not know if the distraction was positive (e.g., curiosity) or negative (e.g., annoyance) for the animals), to dolphins stopping their play to observe visitors and/or interact with them or to our limited behavioural catalogue and the small number of dolphinaria open to the public during the study. Further dolphin welfare studies should consider analysing this parameter.

Dolphins’ play/exploratory behavioural diversity was significantly higher when sensorial, structural, cognitive or mixed enrichment were provided than when no enrichment was present. Enrichment provision aims to increase the display and the variety of play and exploratory behaviours [52]. No enrichment categorised as social was provided during the study. The trainers from each institution were free to choose the type of enrichments they wanted to provide. Sensorial enrichment is often a solitary activity (e.g., a dolphin rubs itself on enrichment, massages itself under a waterjet) whilst cognitive enrichment requires innovative and/or cooperative behaviours with or without the recruitment of partners [69] (i.e., solitary and/or social activity with manipulation of provided enrichment). We then would have hypothesised that cognitive enrichment would create a greater behavioural diversity compared to sensorial enrichment, but this was not evidenced in our study. When enrichment (except nutritional—which serves a practical function and social) was provided, dolphins increased their diversity of play/exploratory behaviours. Playing with objects, but not locomotor play frequency, has been shown to increase with the presence of enrichment in bottlenose dolphins [41]. The frequency of solitary behaviours in odontocetes under professional care is modulated by environmental and social factors [40]. This shows the importance of providing cetaceans in zoological park settings with appropriate and well-defined enrichment programs. In studies on other species, behavioural diversity also increased while enrichment was provided (e.g., big cats [77,78], pigs [79,80,81], bears [82,83], elephants [84] and giant pandas (Ailuropoda melanoleuca) [85]).

### 4.5. Sexual Behavioural Diversity

During the study, no sexual behaviours were observed in only male groups. This result could be due to the seasonal time period of the study [86], the relatively short duration of the study, an observer bias, the limited number of male groups and/or the limited list of behaviours the observers were asked to note (Table 1). Sexual behaviours diversity was significantly lower when only females were present compared to mixed groups. Sexual behaviours are typically intersexual [65]. However, sociosexual behaviours could also be intrasexual [87] and have been previously assessed and observed between all age and sex classes in dolphins [67]. We suggest further studies to include more sociosexual behaviours because sexual behaviours in male groups have been seen in several dolphinaria.

Dolphin groups displayed greater sexual behavioural diversity in groups being separated from conspecifics for longer periods of time (i.e., 5 days) compared to groups where dolphins were always maintained together. “Long term” separated groups were females with their offspring, adult males and mixed-sex groups of juveniles and adults where intrasexual behaviours occurred (e.g., competition) [65] as well as intersexual conflicts (e.g., sexual coercion) [65] and sociosexual behaviours [87]. It is also described that sexual behaviours occur in groups with mothers and juveniles [87,88,89]. Behaviours such as mounting, genital nudging and attempted or actual copulations may also allow young animals to gain experience for future mating opportunities, may promote bonds with other individuals, may be a result of increased hormonal activity during development or may be attributed to a combination of these factors [89]. Sociosexual behaviour frequency has been described to be lower in a group of bottlenose dolphins when animals were separated than when they were altogether [41]. However, in that study, separation was both a social grouping and a sex variable since the separated groups were unisex (one group of females and one group of males). This is a major difference when compared to our study group setting.

### 4.6. Maternal Behavioural Diversity

Maternal behavioural diversity increased with number of individuals and number of mother–calf pairs. We observed maternal behaviours in each group setting with young juveniles. Bottlenose dolphins’ maternal behaviours depend on the calves’ age and on mothers that show different maternal styles to raise their offspring [76], and all have various personalities [60], resulting in greater or lesser maternal behaviours diversity. As expected from previous research performed in the wild and in managed populations, bottlenose dolphin mothers engaged in a variety of maternal care behaviours showing individual variability [90,91,92].

### 4.7. Study Limitations and Further Developments for Dolphin Welfare Assessments

In our study we conducted group focal observations, noting the occurrence of selected behaviours (Table 2). Even if all observers were familiar to their dolphin groups and had previous and numerous experiences in collecting behavioral data for research studies, we cannot avoid interobserver variability. However, the EAAM Welfare Committee intends to set up a dolphin welfare assessment for caretakers to conduct in the facility they work in: this study and others [35,45,46,47] show their feasibility and appropriateness.

We suggest focusing in the future on individuals to study their behavioural diversity according to various parameters known to affect their welfare (e.g., noise/social play [40], personality [93], unusual events and social separation [41]). Individuals’ age and sex are also known to contribute to behavioural variability with, for instance, male dolphins being more aggressive than females [66,94] and young dolphins being more extroverted and curious than adults [60]. Since group behavioural diversity tends to rise with the increasing number of group members, conducting individual behavioural diversity studies would be a valuable addition. 

We also suggest recording the duration and number of occurrences since some behavioural events might be displayed several times in a row and/or last longer than others. For instance, a study on beluga whales showed that young individuals enrich the behavioural repertoire of several adult belugas by decreasing their percentage of time spent in solitary swims and increasing their percentage of time spent in play behaviours and social interactions [95]. In our study, we demonstrated that young dolphins increased the overall groups’ behavioural diversity, play/exploratory behavioural diversity and agonistic behavioural diversity. However, since we missed information on the individuals involved and the nature, duration and frequencies of those behaviours, we cannot make conclusions about their roles in the animals’ welfare. Finally, we also suggest extending the list of behaviours to consider when conducting individual behavioural diversity research. This would have to be tested in order to see if one observer could collect all the targeted behaviours.

Considering the factors which could have some relevance in the establishment of a dolphin welfare assessment methodology, according to a previous study, the factor of time of day [40] impacted dolphins’ affiliative behavioural diversity, but the dolphins’ activity right before and right after the observation [37] did not seem to influence the dolphin groups’ behavioural diversity. This last result differs from a previous study [37], suggesting the need for further research on this subject. Combining aerial and underwater observations increases the chance to observe agonistic and play/exploratory behaviours diversity within a group of dolphins. The combined observations might also prevent some errors due to water turbidity, limited/smaller field view, animals swimming deep in the water column and not being visible from the surface and animal movement between different pools. These factors do not directly impact dolphin welfare; however, they are relevant to consider when setting up an appropriate methodology to assess the animals’ welfare.

We also showed that social grouping (i.e., “long term” separation or all together) and social composition (i.e., presence of a leading male) impacted sexual behavioural diversity and agonistic behavioural diversity, respectively. Dolphin age is also a parameter to consider since we found that the presence of juveniles with adults increased agonistic behavioural and play/exploratory behavioural diversity. Sex composition of the groups is also worth considering. Mixed-sex groups compared to only females increased affiliative and sexual behavioural diversity, while both sexes or only males increased agonistic and play/exploratory behavioural diversity. Finally, in female groups, maternal behavioural diversity increased with the number of mother–calf pairs. Finally, we noticed that when enrichment was provided, greater group play/exploratory behavioural diversity was seen compared to when there was none, and the provision of nutritional enrichment increased agonistic behavioural diversity compared to cognitive enrichment.

## 5. Conclusions

Our study showed the appropriateness and feasibility of performing dolphin group behavioural diversity assessments, and it revealed impacting factors that would need to be considered in future dolphin welfare assessments. We divided dolphin group behavioural diversity into six behavioural categories: affiliative, agonistic, solitary, play/exploration, sexual and maternal, and each of the categories was impacted by different factors (i.e., time of day, number of individuals, group age composition, social structure within the group, presence or absence of the leading male, enrichment provision, presence of the visitors and number of mother–calf pairs). We suggest using group behavioural studies combined with other resource- and animal-based measures when assessing cetaceans’ welfare. Behavioural repertoire richness is often considered to be a positive welfare indicator. We strongly believe that behavioural evaluations and measurements could be applied routinely in cetaceans under professional care to promote good welfare. Having behavioural diversity data regularly monitored could improve an understanding of the behavioural diversity in bottlenose dolphins and their welfare.

## Figures and Tables

**Table 1:** Selected behaviours with their corresponding definition grouped into functional categories.

**Affiliative Behaviours (N = 9)**	
Approaching	Dolphin swims towards a conspecific
Contact swimming	Two or more dolphins are swimming close to each other with a part of their body in contact
Petting/Rubbing	Dolphin rubs its pectoral fin or fluke through an active movement with a conspecific’s pectoral fin or fluke; or fluke to fluke rubbing
Slow group swimming	Several dolphin swim together; slow speed
Synchronous swimming	Two or more dolphins swimming more or less close to each other with synchronised swimming movements
Synchronous breathing	Two or more dolphins breathe in unison
Nibbling	Dolphin nibbles conspecific’s body, usually the fluke
Follow	Dolphin follows another dolphin
Nudge	Dolphin pushes rostrum on another dolphin’s body part
**Agonistic/aggressive behaviour (N = 9)**	
Bite/Rake	Dolphin bites or rakes teeth on another dolphin(s)
Chase	Dolphin follows a conspecific rapidly and intensively
Fast group swimming	At least three dolphins swim in the same direction with a distance of less than one body length between them
Pivot dive	Dolphin briefly leaps out of the water re-entering face first, often during chasing between individuals
Side mounting	Dolphin side mounts or is side mounted by other dolphin/dolphins
Slapping behaviour	Dolphin strikes another dolphin with its head or fluke
Hit/Bump	Dolphin charges into another dolphin using its rostrum or flank in a quick manner
Jaw clapping	Dolphin opens and shuts its jaws rapidly once or consecutively; a loud clapping sound is made
Leaving	Dolphin swims rapidly away from a conspecific
**Sexual behaviour (N = 5)**	
Erection	Male dolphin shows penis out of the genital slit
Genital inspection	Dolphin inspects the genital region of a conspecific and emits a burst pulsed sound; no physical contact is observed
Genital rubbing	Dolphin rubs its genital area on conspecifics
Mating	Two dolphins in ventral contact with intromission observed
Penis insertion	Dolphin inserts its penis into blow hole/anus of a male or female conspecific, but not in its genital area
**Maternal behaviour (N = 5)**	
Nurturant behaviour	A mother carries its calf away from danger
Bumping/genitals	The calf swims underneath its mother and bumps several times into her ventral/genital areas
Nursing	Calf takes milk from its mother
Echelon swimming	Calf is in close proximity of its mother’s mid-lateral flank
Infant swimming	Calf swims underneath its mothers’ peduncle
**Solitary behaviours (N = 19)**	
Arching	Dolphin bends head and tail ventrally
Belly up swimming	Dolphin swims ventral side up for more than five seconds
Bubbles	Dolphin expels air from its blowhole, forming a line of tiny bubbles
Carrying objects	Dolphin carries objects with/on body parts
Circular swimming	Dolphin swims in clockwise or counterclockwise direction in large and regular circles
Fast solitary swimming	Dolphin swims fast, making waves on the surface, or making riddles on its skin
Fluke out of the water	Dolphin hangs vertically in the water, head downward, the tail and the peduncle protruding above water
Head-up swim	Dolphin has its eyes, or the entire head, above the water surface while swimming slowly forward, toward the point of interest
Jumping	Dolphin jumps with a high curvature between head and body; the dolphin jumps and lands in the same place and checks the surrounding environment; the jump is higher than longer
Logging	Dolphin floats at the surface, back and dorsal fin above water and may drift or remains motionless while logging
Looking/surface	Dolphin floats sideways at the surface with one eye above the water to observe; this behaviour may be brief or prolonged, and the dolphin may drift
Regurgitating	Dolphin brings swallowed food up again to the mouth
Resting	Dolphin rests motionless, breathing regularly or it swims slowly and steadily
Rolling	Dolphin’s body is rotated 360° on the longitudinal axis to either side of the dolphin
Rubbing/habitat	Dolphin rubs its body on pool walls, gate or other parts of the environment.
Side swimming	Forward progress in a 90-degree rotation from the dorsal position, orienting one pectoral fin upward and the other downward
Slow solitary swimming	Dolphin swims alone; slow speed
Spy-hopping	Dolphin is vertical in the water and propels itself out vertically usually as far as the pectoral fins, with the eyes directed to a point above the water surface, and then descends again vertically; often the movement can be repeated several times consecutively
Vertical standing	Dolphin hangs/suspends itself vertically with its head up or down in mid-water
**Play/explorative behaviours (N = 8)**	
Exploration	Dolphin investigates or explores habitat (gates, sides and bottom of the pool) or non-enrichment objects (such as tree leaves) by closely looking at them or touching them
Exploration/enrichment	Dolphin investigates or explores enrichment objects by closely looking or touching them
Social play	Dolphin plays with two or more dolphins (including social bubble play)
Solitary play	One dolphin plays (including solitary bubble play)
Object circle	Dolphin begins swimming around an object in wide circles, in other words the object is now included in the swimming circle
Play with object	Dolphin carries a human-made object by using its rostrum, the fins or the melon, the fluke, the mouth, passing and slightly touching an object, balancing/dribbling/catching/throwing and catching/pushing or pulling an object with its rostrum, pressing it under water/rolling it on the bottom of the pool by using the rostrum or the body and holding it in the rostrum while swimming
Play with environment	Dolphin touches, rubs, scratches on, pulls, pushes, splashes, etc., on environmental items
Rough play	Two or more dolphins display intense plays, might include biting, chasing, pivoting

**Table 2:** Summary of impacts of predictors (top row) on functional behavioural categories (left column). Factors associated with significantly higher behavioural diversity are bolded.

	Time of Day	Observer Location	Number of Individuals	Sex	Age	Social Grouping	Presence of Leading Male	Activity before or after Session	Presence of Visitors	Enrichment
Total behavioural diversity		-	Increased with number of individuals	-	Juveniles + adults/adults	-	In absence/in presence	-	-	-
Affiliative behaviours diversity	Morning Midday	-			-	-	-	-	-	-
Agonistic behaviours diversity	-	Under + above observations/above	Increased with number of individuals	Males/females or both sexes	Juveniles + adults/adults	-	In absence/in presence	-	-	Presence/absence Nutritional/cognitive
Solitary behaviours diversity	-	Above water > under or both	-	-	-	-	-	-	-	-
Play/exploratory behaviours diversity	-	Under + above water/under or above water	-	Males or both sexes/females	Juveniles + adults/adults	-	-	-	In absence/in presence	Presence/absence Sensorial, structural, cognitive, mixed.
Sexual behaviours diversity	-	-	-	Both sexes/females	-	Separation/together	-	-	-	-
Maternal behaviours diversity	-	-	Increased with number of individuals			Number of mother-calf pairs	-	-	-	-

## Data Availability

The datasets generated for this study will not be made publicly available. The data is owned by the owners of the animals, but it would be available with their permission.
